# The Dixon method in musculoskeletal MRI: from fat-sensitive to fat-specific imaging

**DOI:** 10.1007/s00256-021-03950-1

**Published:** 2021-12-20

**Authors:** Patrick Omoumi

**Affiliations:** Department of Radiology, Lausanne University Hospital and University of Lausanne, Bugnon 46, Lausanne, CH-1011 Switzerland

## Introduction

In recent years, there has been a growing interest in the use of the Dixon method in a variety of musculoskeletal MRI applications [1–3]. While it has long been utilized in association with gradient-echo sequences, the Dixon method is now also available in association with spin-echo sequences, which form the cornerstone of most musculoskeletal MRI protocols, thereby opening the door to a wide range of applications and its more widespread use in routine practice [1–3]. This perspective paper reviews a few general principles on the interpretation of musculoskeletal MRI, as well as how these principles are influenced by the use of fat-only images generated from a Dixon sequence. To illustrate these principles, we will mainly focus on bone marrow imaging, in which fat plays a key role. In the rest of this paper, all references to sequences correspond specifically to spin-echo pulse sequences (mainly fast/turbo spin-echo (FSE/TSE)) unless otherwise specified.

## Fluid- and fat-sensitive sequences

The so-called fluid-sensitive and fat-sensitive sequences form the basis of musculoskeletal magnetic resonance imaging (MRI) protocols as they allow for the detection and characterization of the two main sources of signal in MRI of the musculoskeletal system, water and fat, respectively.

Fluid-sensitive sequences used in routine clinical practice typically include proton-density-, intermediate-, and T2-weighted sequences. Most pathological processes are associated with increased T2 values due to increased water content, therefore appearing as areas of increased signal intensity (SI) on fluid-sensitive sequences. The sensitivity to detect pathology is further increased by suppressing the signal of fat in fluid-sensitive sequences. Of note, the longer the echo time, the more specific fluid-sensitive sequences are to the signal of liquids.

In clinical practice, “fat-sensitive” sequences have long been limited to spin echo-based T1-weighted (T1w) sequences; they are particularly useful in MRI of the normal adult bone marrow, of which fat is a major component. Indeed, normal bone marrow is always characterized by the presence of fat, which is found both in yellow and red marrow in varying percentages (80% and 40% fat content, respectively) [4]. In bone marrow tumors, normal bone marrow and notably its fatty content is replaced by the proliferation of tumoral cells and other tissue components. Therefore, these bone marrow tumors are referred to as “bone marrow-replacing lesions” and generally show low SI relative to the normal bone marrow on fat-sensitive sequences.

It is important to note that some normal variants of bone marrow (including red marrow islands and focal nodular marrow hyperplasia), as well as some signal abnormality patterns such as bone marrow edema-like signal (usually non-neoplastic in nature), are also hypointense relative to normal bone marrow on T1w sequences [5, 6]. However, because these non-bone marrow-replacing lesions usually contain fat, their SI is not quite as low as that of bone marrow-replacing lesions. In order to characterize these signal abnormalities as benign, it is useful to compare their SI on T1w sequences with that of adjacent muscles or normal intervertebral discs. Due to their preserved fat content, the SI of non-bone marrow-replacing lesions should be higher than that of these internal standards [5–7]. The interpretation of a lesion’s SI by comparison to that of adjacent muscle or discs can, however, be problematic since the latter may present an altered signal itself if pathological (for instance in the case of muscular fatty infiltration or disc degeneration).

## The Dixon method

Recently, the Dixon method has gained interest in musculoskeletal imaging. It consists in the acquisition of in-phase and out-of-phase images, from which water-only (WO) and fat-only (FO) images are reconstructed, producing four sets of images (or more) per acquisition [1, 8]. It has been used primarily as a fat suppression technique because it provides fat-suppressed images (corresponding to WO images) that are more robust against field inhomogeneities than chemical shift selective (CHESS) techniques, while presenting a higher signal-to-noise ratio than short-tau inversion recovery (STIR) techniques [9, 10]. The Dixon method is therefore suitable for large field-of-view applications such as spinal imaging or imaging of the extremities. It has been widely used in association with fluid-sensitive sequences with the added advantage of providing both fat-suppressed and non-fat-suppressed images in a single acquisition.

Authors have subsequently taken advantage of the FO images provided by the Dixon method, and successfully used them as a novel type of fat-sensitive sequence able to replace the information provided by T1w sequences on bone marrow fat content [3, 11–14]. As with T1w sequences, areas presenting increased fat content such as fat islands, hemangiomas, Modic type 2 changes, or fatty lesions related to chronic axial spondyloarthritis show higher SI on FO images, while areas of decreased fat content show lower SI compared to normal marrow. A single fluid-sensitive Dixon sequence therefore may provide the range of contrasts required for the imaging of bone marrow, including non-fat-suppressed fluid-sensitive, fat-suppressed fluid-sensitive, and fat-sensitive images. Of note, non-fat-suppressed T2-weighted sequences are particularly useful to: (1) characterize hyperplastic red marrow, which typically demonstrates lower SI than marrow fat on these sequences [6], and (2) provide the morphological information (e.g., depiction of vertebral body contours in spine imaging), usually provided by T1w sequences. Therefore, the use of a single fluid-sensitive Dixon sequence has led to the simplification and shortening of acquisition protocols in a variety of applications [11–13].

## Not only fat-sensitive, but also fat-specific images

FO images are in fact not only sensitive, but also specific to the signal of fat. In other words, fat is by design the only source of signal on FO images. While T1w sequences are sensitive to the signal of fat as well as all other short T1 substances, FO images selectively show the signal from fat. This has several implications. First, the interpretation of FO images is more straightforward than that of T1w sequences. This is due to the fact that bone marrow-replacing lesions such as neoplastic tumors will practically be void of signal on FO images and, in contrast to the interpretation of T1w sequences, there is no need to compare the lesion SI to that of skeletal muscle or discs in order to determine whether the signal characteristics fulfill the criteria for a bone marrow-replacing lesion. Second, FO images are not subject to potential false negatives in detecting marrow-replacing lesions, as would be the case with T1w images for lesions containing short T1 substances other than fat, such as melanin, methemoglobin, proteinaceous fluid, or paramagnetic substances. Such lesions would be spontaneously high in SI on T1w sequences, hereby decreasing their contrast with normal bone marrow, possibly hindering their detection. Metastases of melanoma or hemorrhagic lesions, for example, classic pitfalls of T1w imaging, demonstrate the same aspect of signal void on FO images as other bone marrow-replacing lesions. Third, the specificity of FO images to the signal of fat lends to a higher contrast-to-noise ratio as compared to T1w sequences, leading to increased lesion conspicuity. This has been evidenced in the context of spinal metastases, multiple myeloma, and periarticular fat deposition in patients with chronic sacroiliitis [11, 13, 15].

**Fig. 1:**
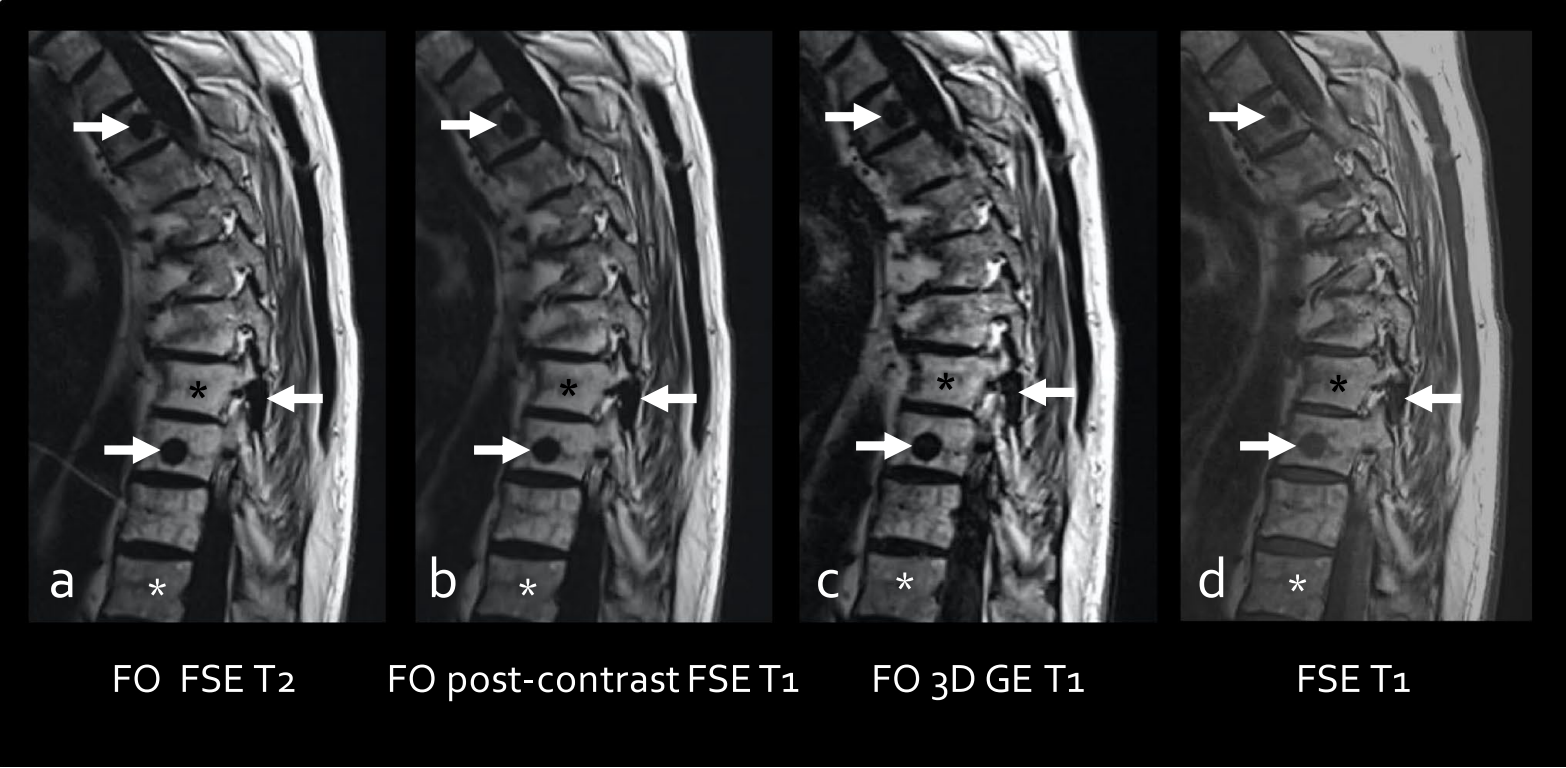
64-year-old male with a history of breast carcinoma treated with surgery, chemo- and radiotherapy. Fat only (FO) (**a**–**c**) and FSE T1-weighted (**d**) images show multiple metastases (arrows) presenting as signal void on FO images. FO images from a FSE T2-weighted (**a**), post-contrast FSE T1-weighed (**b**), and post-contrast 3D gradient-echo T1-weighted sequences (**c**) are almost superimposable. Note the higher contrast between the lesions (arrows) and surrounding tissues, as well as between fatty marrow (post-radiotherapy) (black asterisk) and red marrow (white asterisk) on FO images (**a-c)** compared to the FSE T1-weighted image (**d**). Based on the data from the literature, our spine protocols in the sagittal plane (workup of metastases, degenerative spine, spondyloarthritis) have been reduced to a single FSE/TSE fluid-sensitive Dixon sequence providing four sets of images [3, 11–14]

Finally, the specificity of FO images to the signal of fat leads to some further interesting applications. First, FO images may be acquired in association with any type of sequence weighting or pulse sequence (i.e. spin-echo or gradient-echo) to provide information on the fat content of a certain tissue. For example, the Dixon method may be utilized with post-contrast T1w sequences in order to provide information on bone marrow or tumoral fat content despite the injection of intravenous contrast material (which does not interfere with the signal on fat-specific sequences). Indeed, FO images generated from a Dixon method will have the same aspect, whether the sequence is a fluid-sensitive sequence, a non-contrast T1-, or post-contrast T1-sequence or even a gradient-echo sequence (Fig. 1). As another practical application, gradient-echo Dixon sequences routinely performed in liver imaging may be used to reconstruct FO images in order to detect and characterize bone marrow-replacing lesions in the nearby skeleton.

## Not only qualitative, but also quantitative information

One of the advantages of using the Dixon method is that the same sequence used for qualitative assessment in routine practice may also provide quantitative information on the relative amount of fat and water [16, 17]. Indeed, spin-echo-based Dixon sequences can be used to quantify signal drop between in-phase and out-of-phase images or map the fat fraction. Previously, “conventional” chemical shift imaging performed with gradient-echo sequences had been considered to be a useful adjunct for the characterization of bone marrow lesions. Using that technique, a signal drop of less than 20% is usually indicative of a significant decrease in fatty content and may be used as an additional criterion for the diagnosis of bone marrow-replacing lesions [18, 19]. The same threshold may be applied when using spin-echo-based Dixon sequences [16]. False positives include areas of pure fat or pure water, easily recognizable on WO, FO, and in-phase images.

A signal drop above 20% indicates the presence of both fat and water in the region of interest, which is the case for non-bone marrow-replacing lesions, mostly consisting of benign lesions or variants such as red marrow islands, focal nodular marrow hyperplasia, and inflammatory or posttraumatic bone marrow edema-like signal. In practice, quantitative assessment may be used to complement qualitative assessment and may serve as an additional criterion in the differentiation of benign from malignant bone marrow lesions. The quantitative information is readily available on the same sequence that is used for morphological analysis, and may be helpful in doubtful cases, for example, when there seems to be some intralesional fat SI at qualitative analysis.

## “Pitfalls” to keep in mind while interpreting fat-only images

- First, as discussed above, FO images cannot provide the anatomical information contained in T1w images. Other sets of images, such as in-phase images should be used to retrieve morphological information. Sequence optimization is generally required to find a compromise between acquisition time, which is intrinsically longer with Dixon methods, signal-to-noise ratio, and spatial resolution. Referring to an application specialist is helpful since different Dixon methods exist, depending on the manufacturer and scanner type.
- Second, while FO images perform well in the detection of bone marrow-replacing lesions, additional sequences may be needed for complete lesion characterization. Specifically, the work-up of a newly discovered isolated lesion requires the acquisition of pre- as well as post-contrast T1w sequences to provide information on contrast enhancement or on the presence of other short T1 components such as melanin in case of metastases of melanoma for instance (showing high SI on pre-contrast images).
- Third, previous treatment of bone marrow lesions may induce the appearance of intralesional fat, which could lead to falsely characterizing these lesions as benign (a pitfall that also applies to T1w sequences). In these cases, the clinical context usually is helpful in avoiding misdiagnosis.
- Fourth, reconstruction artifacts, called “swapping artifacts” occasionally occur. They are present on both sets of reconstructed images (WO and FO images) and are usually easily characterized as artifacts by comparison with the in-phase images [1, 20].
- Fifth, it is important to keep in mind that the quantitative assessment is mostly useful in differentiating bone marrow replacing from non-bone marrow-replacing lesions, rather than differentiating benign from malignant lesions. While most non-bone marrow-replacing lesions containing preserved normal marrow fat are benign, some benign tumors may manifest as bone marrow-replacing lesions. The quantitative assessment is therefore a useful adjunct to rather than a replacement of qualitative assessment.
- Finally, some benign lesions associated with increased mineralization such as healing fractures may lack a signal drop on out-of-phase images, which may lead to falsely characterizing them as marrow-replacing lesions [16].

## Conclusion

The use of FO images generated from the Dixon method has changed the way we practice bone marrow imaging, and there are many benefits to applying this technique in oncological imaging. We encourage the readers who are not familiar with this method to use it as a fat suppression technique and experiment with the interpretation of FO images that are provided in the same acquisition. Eventually, the Dixon method may lead to the simplification and shortening of acquisition protocols, while providing many additional benefits, some of which were reviewed here [1].

## Declarations

### Research involving animals

Does not apply.
